# Is mental health in South Africa moving forward?

**DOI:** 10.1192/bji.2023.32

**Published:** 2024-02

**Authors:** Tejil Morar, Jozef Erasmus Breedt, Nokuthula Mdaka, Kagisho Maaroganye, Lesley Robertson

**Affiliations:** 1 Psychiatrist, Department of Psychiatry, School of Clinical Medicine, Faculty of Health Sciences, University of the Witwatersrand, Johannesburg, South Africa. Email: tejil.morar@wits.ac.za; 2 Psychiatric Registrar, Department of Psychiatry, School of Clinical Medicine, Faculty of Health Sciences, University of the Witwatersrand, Johannesburg, South Africa; 3 Psychiatrist, Department of Psychiatry, School of Clinical Medicine, Faculty of Health Sciences, University of the Witwatersrand, Johannesburg, South Africa; 4 Psychiatrist, Department of Psychiatry, School of Clinical Medicine, Faculty of Health Sciences, University of the Witwatersrand, Johannesburg, South Africa; 5 Psychiatrist, Department of Psychiatry, School of Clinical Medicine, Faculty of Health Sciences, University of the Witwatersrand, Johannesburg, South Africa

**Keywords:** Mental health services, SA Mental Health Conference, low- and middle-income countries, mental health policy, South Africa

## Abstract

A landmark South African Mental Health Conference took place in April 2023, marking the first national collaborative conference between government and mental health professionals. The theme was Join the Movement, and a ‘whole of society’ approach was emphasised, imploring various sectors to collaborate in relieving the country's burden of mental illness. Challenges in mental health were raised and possible solutions presented. This article discusses the conference, aspects of psychiatric care in South Africa, South Africa's health system issues and the importance of moving forward measurably.

For the first time in South African history, government collaborated with mental health professionals to host a conference dedicated to mental health. This landmark South African Mental Health Conference (SAMHC) was held in Johannesburg in April 2023.^1^ It signified a milestone in acknowledging the magnitude of mental illness, its impact in South Africa and the need for action, similar to the role of the first South Africa AIDS Conference 11 years ago. The true litmus test in determining the success of the SAMHC venture will lie in how much change will be implemented. But how do we measure this change? And who can be held accountable for it?

The SAMHC comes at an important time in South Africa as common mental illnesses are estimated to have increased three- to seven-fold since the onset of the coronavirus disease 2019 (COVID-19).^1^ In the recent Mental State of the World Report released by Sapien Labs, South Africa ranked second lowest on the Mental Health Quotient and highest in terms of the percentage of those stressed/distressed out of 64 countries.^2^

Factors contributing to the causation and exacerbation of mental illness in South Africa include poverty, unemployment, inequality, violence, gender-based violence and political upheaval (both in the past and currently).^3^ Between 2000 and 2022 the unemployment rate in the country rose from 20.3 to 29.8%.^4^ A multisectoral approach to the aetiology and impact of mental illness in South Africa is needed, requiring participation of the South African Department of Social Development and South African Department of Justice and Constitutional Development, among others.

## Access to health services

The most recent national prevalence study, the South Africa Stress and Health (SASH) study, was conducted almost 20 years ago, in 2004.^5^ The lifetime prevalence of any mental disorder was found to be 30.3%, with anxiety disorders being the most prevalent, at 15.8%. Substance use disorders were next, at 13.3%, followed by mood disorders at 9.8%.^5^ Although the SASH study found a treatment gap of 75%, a national costing study conducted in 2016–2017 found a treatment gap of 91% among those who cannot afford private healthcare.^6^ Current epidemiological data are sorely needed if adequate services are to be planned, also noting that socioeconomic factors have worsened for many since 2004.

Problematic access to healthcare services occurs at both a systemic level and an individual level.^1^ At the systemic level, possible solutions include: (a) stratifying plans and policies in a sequential order, (b) determining costs of implementation, (c) indicating responsible persons and timelines for plans, (d) setting up monitoring teams for each step of the entire implementation programme, (e) training and retraining healthcare managers and health authorities in mental health-related matters, as these managers are the persons to turn strategy into action by providing resources for the action and monitoring it, and finally (f) providing feedback to higher authorities on the success or failure of the strategy. On an individual level, internalised and externalised stigma play a major role, and this was correctly highlighted numerous times during the SAMHC.^1^

## The current health system

South Africa currently employs a two-tier health system: a government-funded public sector and private funders:^7^ 84% of the population use the government-funded public sector and 16% access private care using private funding mechanisms. National Health Insurance (NHI) seeks to unify the health system through pooling of private and public funds.^7^ The recently passed NHI Bill was put forward during the conference as a possible solution to inadequate access to healthcare services experienced by most of the South African population.^1^ However, the South African Medical Association (SAMA) has expressed major concerns regarding NHI.^8^ Issues such as contracting units for primary healthcare and benefit packages and reimbursement models have not been finalised.^8^

Life Esidimeni, a private healthcare provider, provides long-term psychiatric facilities to patients in the public sector. The ‘Life Esidimeni tragedy’ occurred after the Gauteng Health Department terminated its contract with Life Esidimeni Health Care Centre in 2016. Patients were transferred rapidly to unlicensed non-governmental organisations, which were poorly equipped to take care of people with severe mental illness. The transfer was done under the disguise of de-institutionalisation, but poor planning, mismanagement (including financial) and unaccountability resulted in 144 people with mental illness losing their lives.^9^ It would be naive to expect that an increased pool of funding alone will improve access to mental healthcare in South Africa. The efficiency, effectiveness and resilience of mental health services is imperative and cannot be obtained without transparency and accountability.^8^

Facilities providing mental health services in South Africa can be divided into healthcare facilities (hospitals, specialised mental health clinics, etc.), community responses (support groups, counselling, etc.) and institutional responses (employers, higher education institutions, etc.).^3^ There was an emphasis on community-based services led by people with lived experience during the conference.^1^ However, people with lived experience are not well represented in decision-making bodies and healthcare planning committees, or in community forums in South Africa.

Community engagement has been shown to potentially contribute to more culturally competent services and personal empowerment of recipients of care.^9^ South African culture includes *Ubuntu* (humanity), traditional African healing and ancestral callings, all of which intersect with psychiatry. In addition, community participation promotes a community-led approach to alleviating some of the social determinants of mental ill health described above.^9^ To improve access to mental healthcare and recovery for people with mental health conditions community-based interventions with community engagement need to be strengthened.

## Technology

The increased accessibility to mental healthcare through digital platforms is promising, although it is challenged by provision of infrastructure for access in low- and middle-income countries, where issues such as food insecurity, housing instability and job scarcity often prevail. During the COVID-19 pandemic, South Africa utilised short message services (SMS), mobile applications and WhatsApp communication to disseminate information and connect patients to healthcare services. However, these interventions highlighted the digital divide between urban and rural settings. Financial constraints stand out as a significant limitation in ensuring equitable roll-out of digital medicine.^10^

The bridging of this digital divide necessitates enhanced infrastructure, focused on internet connectivity and power supply. This requires political and budgetary support while fostering collaboration between public and private sectors. Providing training to community healthcare workers in utilising digital health solutions and raising awareness among rural communities is crucial. The degree of response and utilisation of these services depends on effective distribution of knowledge and skills.^10^

## Human resources

Mental health service delivery in South Africa is severely affected by human resource challenges. There is an average of 0.31 psychiatrists per 100 000 population in the South African state sector, with an unequal distribution between rural and urban areas.^6^ Some predominantly rural provinces have only 0.08 psychiatrists per 100 000 population in the state sector. There is also a critical shortage of child psychiatrists, with only three of the nine provinces having any child psychiatrists in the state sector.^6^ Around 50% of state hospitals offering psychiatric care do not have a psychiatrist and 30% have no clinical psychologists.^3^

This burden on available healthcare workers is concerning and a large portion of the conference was dedicated to the mental well-being of healthcare workers in South Africa.

Interventions in South Africa for healthcare providers who require assistance for their mental health include the national Healthcare Workers Care Network (HWCN).^1^ Dr Antoinette Miric, co-founder of the HWCN, highlighted at the conference that healthcare workers are particularly vulnerable to workplace stress, and this came to light during the recent COVID-19 pandemic.^1^ Future recommendations include improvement in environment (safety, hygiene, water, electricity), proactive leadership in improving working conditions, gratitude from service users, easy access to confidential counsellors and employee assistance programmes, empowered team leaders, decreased stigma and improved access to care.^1^ Initiatives like the HWCN offer healthcare workers a portal to gain access to mental healthcare.

However, some concerns exist. Healthcare practitioners, particularly medical doctors, are notoriously reluctant to seek help or disclose their mental state when mentally unwell,^11^ and this is likely applicable to other healthcare providers. Reasons include stigma and fears of being labelled, confidentiality and lack of understanding of services available.^11^ It then raises the question of whether there will be an uptake of the services mentioned above. Another concern is the lack of monitoring using robust data, to optimise the strengths of these interventions and minimise unhelpful aspects.

## Policy

Legislation and policy in South Africa, such as the Mental Health Care Act No. 17 of 2002 and the previous National Mental Health Policy Framework and Strategic Plan 2013–2020, is progressive, but implementation has been criticised.

The National Mental Health Policy Framework and Strategic Plan 2023–2030 (the policy) has an ambitious vision to achieve ‘comprehensive, high quality, integrated mental health promotion, prevention, care, treatment and rehabilitation for all in South Africa by 2030’.^12^ The intervention pyramid for the organisation of services is the same as in the previous (2013–2020) policy, and the strategic plan almost identical. Notable differences are:

- a greater understanding of imbalance and inequity in current mental health services, largely derived from the national costing survey conducted by Docrat et al (2019)^6^
- the clearer directives on strengthening community-based and integrated services, with a gradual downscaling of psychiatric hospitals, as recommended in the World Health Organization's (WHO) Mental Health Report of 2022^13^
- a section on mechanisms for monitoring implementation, with a list of 18 priority mental health indicators.

Poor implementation of the previous policy was evident in the conference sessions, particularly with respect to youth.^1^ The question of how to provide accessible, quality care to children and adolescents was pervasive. A suggestion from one attendee was to ‘flatten the intervention pyramid and place all the specialists in the community’.

Although this suggestion is consistent with the WHO Mental Health Report, the emphasis on specialist care does not appear to address the social determinants of poor mental health or the need for multi-sectoral collaboration. However, the idea does resonate with the concept of community psychiatry,^14^ which incorporates multidisciplinary person-centred care within the person's social and cultural context, inter-sectoral collaboration on individual and family mental health, as well as outreach engagement with local society in developing programmes for primary prevention and community well-being.

Funding towards policy implementation was missing from the conference agenda, and there was no promise of dedicated funding of the mental health programme. How the non-healthcare sectors will fund their obligations was also unclear. The affordability of a successful mental health programme in the face of South Africa's social ills is also questionable. Nevertheless, detailed implementation guidelines with defined outcomes for both the healthcare and non-healthcare sectors could go a long way in ensuring funds are made available for action.

## Conclusion

The theme of the conference was Join the Movement, which echoes a statement by the WHO: ‘Change is not happening fast enough. And the story of mental health is one of need and neglect’.^1^ South Africa has now had its first mental health conference, raising a broad range of issues and placing many potential options for change. Advancing information systems, transparent management and monitoring of indicators are imperative in moving mental health in South Africa forward.
